# Rituximab for auto-immune alveolar proteinosis, a real life cohort study

**DOI:** 10.1186/s12931-018-0780-5

**Published:** 2018-04-25

**Authors:** Berenice Soyez, Raphael Borie, Cedric Menard, Jacques Cadranel, Leonidas Chavez, Vincent Cottin, Emmanuel Gomez, Sylvain Marchand-Adam, Sylvie Leroy, Jean-Marc Naccache, Hilario Nunes, Martine Reynaud-Gaubert, Laurent Savale, Abdellatif Tazi, Lidwine Wemeau-Stervinou, Marie-Pierre Debray, Bruno Crestani

**Affiliations:** 1 Service de Pneumologie A, DHU FIRE, centre de référence constitutif des maladies pulmonaires rares, Hôpital Bichat, APHP, 46 rue Henri Huchard 75877 Paris CEDEX, 18 Paris, France; 2 OrphaLung, Lyon, France; 3 Service de Pneumologie, Hôpital de la Pitié Salpetrière, APHP, Paris, France; 4 Service de Radiologie, Hôpital Bichat, APHP, Paris, France; 5 Service d’Immunologie, Thérapie Cellulaire et Hématopoïèse, CHU Pontchaillou, Rennes, France; 6 Service de Pneumologie, Centre de référence constitutif des maladies pulmonaires rares, Hôpital Tenon, APHP, Paris, France; 7 Service de Pneumologie, Centre de compétences des maladies pulmonaires rares, CHU Grenoble-Alpes, Grenoble, France; 8 Service de Pneumologie, Centre national de référence des maladies pulmonaires rares, Hôpital Louis Pradel, Université Claude Bernard Lyon 1, Lyon, France; 9 Service de Pneumologie, Centre de compétences des maladies pulmonaires rares CHRU Nancy, Nancy, France; 10 Service de Pneumologie, Centre de compétences des maladies pulmonaires raresCHRU de Tours, Tours, France; 11 FHU Oncoage, Service de Pneumologie, Centre de compétence des maladies pulmonaires rares, Université Côte d’Azur, CHU de Nice, Nice, France; 12 Service de Pneumologie, Centre de référence constitutif des maladies pulmonaires rares, Hôpital Avicenne, APHP, Bobigny, France; 13 Service de Pneumologie, Centre de compétence des maladies pulmonaires rares, Hôpital Nord, Marseille, France; 14 Service de Pneumologie, Centre de référence de l’hypertension pulmonaire, Hôpital Bicêtre, APHP, Le Kremlin Bicêtre, France; 15 Service de Pneumologie, Hôpital Saint-Louis, APHP, Paris, France; 16 Service de Pneumologie, Centre de référence constitutif des maladies pulmonaires rares, CHRU de Lille, Lille, France; 17 INSERM, Unité 1152, Université Paris Diderot, Paris, France

**Keywords:** Whole lung lavage, GM-CSF, Surfactant, Interstitial lung disease, Therapy

## Abstract

**Background:**

Whole lung lavage is the current standard therapy for pulmonary alveolar proteinosis (PAP) that is characterized by the alveolar accumulation of surfactant. Rituximab showed promising results in auto-immune PAP (aPAP) related to anti-GM-CSF antibody.

**Methods:**

We aimed to assess efficacy of rituximab in aPAP in real life and all patients with aPAP in France that received rituximab were retrospectively analyzed.

**Results:**

Thirteen patients were included. No patients showed improvement 6 months after treatment, but, 4 patients (30%) presented a significant decrease of alveolar-arterial difference in oxygen after 1 year. One patient received lung transplantation and one patient was lost of follow-up within one year. Although a spontaneous improvement cannot be excluded in these 4 patients, improvement was more frequent in patients naïve to prior specific therapy and with higher level of anti-GM-CSF antibodies evaluated by ELISA. No serious adverse event was evidenced.

**Conclusions:**

These data do not support rituximab as a second line therapy for patients with refractory aPAP.

## Background

Pulmonary alveolar proteinosis (PAP) is a rare disease characterized by deposition of extracellular lipoproteinaceous material within pulmonary alveoli [1]. PAP usually results from failure of clearance of surfactant by alveolar macrophages [2–4]. The most common cause of PAP is autoimmune (in 90% of cases, aPAP) relevant to the presence of anti-GM-CSF (Granulocyte-macrophage colony-stimulating factor) autoantibodies. GM-CSF, a growth factor for granulocytes and monocytes, stimulates the differentiation, the proliferation and the survival of myeloid cells [5]. Anti-GM-CSF antibodies bind with high affinity to GM-CSF thus blocking receptor binding and its specific activity [6–8]. Alveolar macrophages are then no longer able to clear alveolar surfactant, and also have a poor local anti-infectious activity [9]. The pathogenicity of anti-GM-CSF antibodies has been proven by the development of PAP after administration of anti-GM-CSF antibodies to non human primates [10, 11].

Whole lung lavage is the current standard therapy for PAP and has been shown to improve the prognosis. Whole lung lavage is effective in almost 85% of patients [12]. However, whole lung lavage is associated with adverse effects such as infections, fever, convulsions, pneumothorax, pleural effusion, hypoxemia or even death. Moreover, 15% of patients do not improve and 10% of patients need repeated whole lung lavage [13–19]. Corticosteroids are not effective and may be associated with an increased risk of opportunistic infection [20]. Inhaled or sub-cutaneous GM-CSF is currently investigated as an alternative to therapeutic lung lavage (NCT02702180). Retrospective and prospective data suggest a persistent improvement in 48% to 100% of the cases [21–23]. Rituximab, a monoclonal antibody directed against the CD20 antigen, has been shown to be effective in several autoimmune diseases such as rheumatoid arthritis or granulomatosis with polyangiitis [24–26]. Rituximab has been reported to improve aPAP in 3 isolated cases and in 7/9 patients of a prospective series [27–30]. While, rituximab showed early promising results and appears to be an interesting alternative treatment, we aimed to evaluate its efficacy on aPAP in real life.

## Methods

### Study design

We retrospectively identified all French patients with aPAP who received rituximab between 2007 and 2014, from a mailing list to the French network of competence and reference centers for rare pulmonary diseases. In France, almost all of the patients with rare lung diseases, and particularly with aPAP, are referred to one of the competence/reference center.

All patients gave informed consent for data collection and the use of rituximab beyond marketing authorization. The Institutional Review Board of the French-learned society for respiratory medicine (Société de Pneumologie de Langue Française) approved this retrospective study (CEPRO 2012–016).

### Inclusion criteria

The patients were included in the study if 1) they had evidence for PAP as assessed by bronchoalveolar lavage (BAL), transbronchial biopsy or open lung biopsy; and 2) an anti-GM-CSF antibody was detected; and 3) they had received at least one dose of rituximab.

### Data collection

The clinical charts of the patients were reviewed and the following data were collected by use of a standardized and anonymous collection form: at the first rituximab infusion (day 0 or baseline), and at day 14, at months 3 (M3), M6, M9, M12. We collected clinical data, standard biological tests results, anti-GM-CSF antibodies titer, chest high-resolution computed tomography (CT), and results of lung function tests, and blood gases when available.

### Anti-GM-CSF titer

GM-CSF neutralizing activity of serum was evaluated in a functional bioassay [4]. Briefly, the GM-CSF-dependent TF-1 cell line was incubated with serial serum dilutions in the presence of 1 ng/mL recombinant GM-CSF (Cellgenix, Germany). After 48 h incubation, ^3^H-thymidine was added for further 8 h. Thymidine uptake by TF-1 cells was assessed by quantification of the radioactivity with a scintillation counter. The GM-CSF antibody titer was calculated as the inverse of the serum dilution blocking 50% of the maximum TF-1 proliferation.

In addition, in some patients, the serum GM-CSF antibody concentration was assessed by ELISA as previously described [4, 31, 32], with some modifications. Briefly, 96-wells Maxisorp® plates (Corning, USA) were coated overnight with 1 μg/mL GM-CSF in phosphate buffer saline (PBS) then washed with PBS-0.05% Tween and blocked with PBS-1% bovine serum albumin for 2 h. After 3 washing steps, diluted sera were added to the wells and incubated for 40 min at room temperature. Then wells were washed 5 times with PBS-0.1% Tween and bound GM-CSF antibodies were detected by anti-human IgG biotinylated antibody (BD Pharmingen, USA) combined to streptavidin conjugated to horseradish peroxidase. Chromogenic substrate solution (TMB Substrate Reagent, BD Pharmingen) was added. Optical absorbance was measured at 450 nm. Anti-GM-CSF concentration was calculated using a standard calibration curve obtained with human polyclonal intravenous immunoglobulins.

All assays were done in one single expert center. Functional assays were done at time of blood taking, while ELISA measurements were done for all samples at the same time.

### Chest CT analysis

Chest CT obtained before (baseline), or 6 months and 12 months after rituximab infusion, were anonymously analyzed by an experienced thoracic radiologist (MPD), patient by patient, blinded to the time from treatment. The extent of CT reticulations and ground-glass opacities was scored between 6 and 30 by summing the grades of 6 pulmonary zones (adapted from [33]). The more extended the CT lesions, the higher the score.

### Assessments

Improvement after rituximab therapy was defined by a decrease ≥10 mmHg of the alveolar–arterial gradient at rest (D(A-a)O_2_) as compared to baseline. A deterioration was defined by an increase ≥10 mmHg of the D(A-a)O_2_.

Other endpoints considered as significantly improved were: ≥ 10 mmHg increase of arterial partial pressure of oxygen (PaO_2_), ≥ 10% increase in diffusing capacity of the lung for carbon monoxide (DLCO) or absolute value of vital capacity (VC), ≥ 50% decrease in GM-CSF antibody level, ≥ 3 points decrease in CT-grade. The disease severity score (DSS) was assessed as follow: DSS 1: no symptoms and PaO2 ≥ 70 mmHg, DSS 2: symptomatic and PaO2 ≥ 70 mmHg, DSS 3: PaO2 ≥ 60 mmHg and < 70 mmHg, DSS 4: PaO2 ≥ 50 mmHg and < 60 mmHg and DSS 5: PaO2 < 50 mmHg [22].

### Statistical analysis

Continuous data are presented as mean (minimum-maximum) and compared by the Mann-Whitney’s test. Categorical data are presented as numbers (%) and compared by the Fisher’s test. All tests were bilateral with a *p* < 0.05 considered as significant. All analyses were performed with the GraphPAd prism software (La Jolla, USA).

## Results

### Patients

We identified a total of 13 patients (9 men) from 11 centers, including one patient previously reported [28] (Table 1). The mean age at aPAP diagnosis was 46 years and the mean time since diagnosis was 28.5 months. Seven patients (54%) were smokers, including 4 active smokers, and 8 patients (62%) had been exposed to inhaled toxics.

**Table 1:** Patients characteristics at inclusion

Patients	1	2	3	4	5	6	7	8	9	10	11	12	13
Age (year)	44	56	50	30	53	41	49	44	59	41	51	49	45
Gender	M	M	M	F	M	F	M	F	F	M	M	M	M
Duration of PAP (months)	38	12	12	29	64	50	6	32	9	15	45	20	51
Smoking status	Never	Never	Current	Current	Never	Never	Never	Never	Ex	Current	Ex	Ex	Current
Inhaled toxic	No	No	Yes	Yes	Yes	No	Yes	No	Yes	Yes	Yes	Yes	No
Previous therapy	No	WLL GM-CSF	WLL	WLL	WLL GM-CSF	WLL GM-CSF	No	WLL GM-CSF	WLL	WLL	WLL GM-CSF	WLL	No
Initial Improvement with WLL	Np	No	No	Yes	Yes	Yes	Np	Yes	No	No	No	Yes	Np
Aggravation in the 3 month before RTX	Yes	No	Yes	Yes	No	Yes	Yes	No	Yes	Yes	Yes	Yes	Yes
Disease severity score	3	2	2	2	2	4	3	4	2	2	3	2	3
Anti GM-CSF titer before RTX	10,000	480	400	700	1000	200	400	400	40	300	300	800	4000
D(a-A)O2													
At RTX injection	36	36	40	38	31	50	46	57	37	35	47	35	40
At M12	20	32	60	51	30	Na	35	60	38	Na	15	26	27

*PAP* Pulmonary alveolar proteinosis, *M* Male, *F* Female, *WLL* Whole Lung lavage, *RTX* Rituximab, *Na* Not available, *Np* not performed

For patients without dosage at rituximab injection, the last available concentration of anti-GM-CSF is reported

Prior to rituximab, 10 patients (77%) were treated with whole lung lavage, with a mean of 2.8 lavages and a median of 2.0 lavages per patient. No patient received whole lung lavage within 3 months before inclusion. Five patients (38%) had received GM-CSF prior to rituximab, including one who was still treated by inhaled GM-CSF at first infusion of rituximab.

### Rituximab therapy and efficacy

Twelve patients received two infusions of rituximab (1000 mg) (at day 0 and day 14) among whom three received a third infusion at month 6, 9 and 12 respectively. One patient received only one infusion of rituximab (1000 mg) and was lost to follow-up after month 3. One patient was treated with plasma exchange one month after enrollment, and then received a lung transplant. Eleven patients were still followed from M3 (Table 2).

**Table 2:** Characteristics at inclusion and after rituximab

Mean values	At Rituximab injection	After rituximab			*p*-value*
		M3	M6	M12	
Number of patients	13	11	11	11	
D(A-a)O_2_ (mmHg)	40 (31–57)	43 (34–60)	40 (31–56)	35.9 (15–60)	0.19
PaO_2_ (mmHg)	68 (57–82)	63 (55–70)	65 (52–78)	70 (49–92)	0.51
Disease Severity Score	2.7 (2–4)	3.0 (2–4)	2.8 (2–4)	2.3 (1–5)	0.44
VC (%predicted value)	79 (36–100)	78 (61–87)	79 (50–100)	84 (62–102)	0.81
DLCO (% predicted value)	54 (19–92)	46 (30–86)	50 (29–79)	63 (35–97)	0.38
Serum anti-GM-CSF					
ELISA (μg/mL)/Available	99 (18–220)/8	Na	85 (19–187)/7	7.7 (3.1–158)/6	0.19
Functional (titer)/Available	853 (200–4000)/9	Na	831 (30–4000)/11	436 (0–1400)/8	0.21
CT-grade	19.3 (12–28)	Na	19.3 (13–28)	17.3 (6–26)	0.73

*between at rituximab injection and M12, *VC* Vital capacity, *DLCO* Diffusing capacity of the lung for carbon monoxide, *CT* Computed tomography, *D(A-a)O*_*2*_ alveolar–arterial gradient at rest_,_ *na* not available

**Fig. 1:**
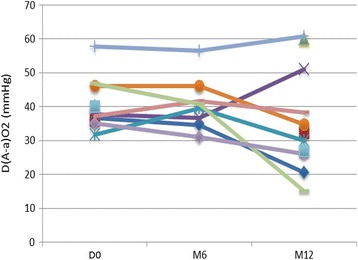
Changes in D(A-a)O_2_ before (D0) and 6 (M6) et 12 (M12) months after rituximab therapy

In the whole population, rituximab was not associated with significant improvement in any of the endpoints at M3, M6 or M12 (Table 2). However when assessed individually some patients did improve (Fig. 1).

At M6 after rituximab infusion, 3 patients deteriorated their lung function and required whole lung lavage or GM-CSF therapy. D(A-a)O_2_, PaO2 or VC was not improved in any patient. DLCO improved in one patient and CT scan improved in two patients.

At M12 after rituximab infusion, a significant improvement of D(A-a)O_2_ was observed in 4/11 patients (36%, none of them had received any additional therapy after rituximab), while D(A-a)O_2_ was stable in 5 patients (45%, including 2 patients who received specific aPAP therapies because of an early deterioration) and decreased in 2 patients (18%). In the improved group, the mean D(A-a)O_2_ decreased from 42.4 mmHg before infusion to 24.3 mmHg at M12 and the mean PaO_2_ get from 64.3 mmHg before infusion to 82.8 mmHg at M12 (Table 3). In parallel, at M12, VC increased in 1/10 patients (10%), DLCO in 5/10 patients (50%), and CT score decreased in 4/9 patients (44%). Three patients had both increased DLCO and D(A-a)O_2_ and decreased CT score.

**Table 3:** Factors associated with therapeutic response in patients evaluated at M12

	Improved (*n* = 4)	Non-improved (*n* = 7)	*p*-value
Age	47 (41–51)	48 (30–59)	0.70
Gender male	4 (100%)	4 (57%)	0.24
Toxics inhalation or significant smoking	3 (75%)	5 (71%)	1.0
Prior treatments to rituximab	1 (25%)	7 (100%)	0.02
Severity			
PaO_2_ (mmHg)	64.3 (63–66)	70.7 (57–82)	0.07
D(A-a)O_2_ (mmHg)	42.4 (36.7–46)	39.4 (31.7–57.7)	0.32
VC (%)	94.3 (85–105)	83.0 (54–95)	0.92
DLCO (%)	49.0 (19–64)	56.9 (38–92)	0.30
CT-grade	17.8 (12–23)	20.1 (14–28)	0.50
Serum anti-GM-CSF			
ELISA (μg/mL)	169.3 (78–220)	58.2 (18–104)	0.03
Functional (titer)	1566.7 (300–4000)	496.7 (200–800)	1.0

*VC* Vital capacity, *DLCO* Diffusing capacity of the lung for carbon monoxide, *CT* Computed tomography, *D(A-a) O*_*2*_ alveolar–arterial gradient at rest

### Evolution of anti-GM-CSF titers

Data were both available at rituximab injection and at M12 for 6 patients evaluated by the functional assay and 4 patients evaluated by ELISA.

Globally, the mean level of anti-GM-CSF antibodies was not significantly modified before and after rituximab, evaluated either by ELISA or by functional assay (Table 2). However, 4 patients (67%) showed a significant decrease (≥ 50%) of anti-GM-CSF antibody titers at M12 as evaluated by the functional assay, among which 2 showed an improvement of D(A-a)O_2_, while 1 patient (25%) showed a significant decrease of anti-GM-CSF antibody titers as evaluated by ELISA, and this patient did not experience improvement of D(A-a)O_2_.

### Characteristics of patients who improved after rituximab

We assessed the characteristics of patients who improved defined by a decrease ≥10 mmHg of the alveolar–arterial gradient at rest (D(A-a)O_2_) at M12 as compared to baseline. The patients who improved were four men aged 45 to 51 years. Three were current or ex-smokers and one of them was exposed to wool dusts and bird feathers. There was no significant difference between improved and non-improved patients on age, gender, smoking history or toxic inhalation, and disease severity at rituximab initiation. Patients who improved had higher serum titers of anti-GM-CSF antibodies evaluated by ELISA, received less specific aPAP therapies prior to rituximab and tended to have lower PaO_2_ at inclusion (Table 3). None of the patients who improved received adjuvant PAP therapy in the 12 months following rituximab infusion.

### Adverse events

No serious adverse events related to rituximab were identified. A cutaneous rash or pruritus occurred after the first rituximab administration in 3 patients, and spontaneously resolved. One patient developed deep-vein thrombosis and one patient complained of sleep and concentration troubles. During follow-up, 2 patients developed viral upper respiratory tract infection and one patient was given a diagnosis of *Mycobacterium avium* airway colonization. No opportunistic infection was observed.

## Discussion

This series is the largest series evaluating rituximab in aPAP. Rituximab was well tolerated and adverse effects were rare and minor. Rituximab was associated with an objective improvement in only 4 of the 13 patients treated (30%) after 12 months. Interestingly, improvement only occurred after 6 months of treatment and was more frequent in patients naive of specific aPAP therapy and with higher level of GM-CSF auto-antibodies evaluated by ELISA.

One prospective clinical trial by Kavuru et al., which included 9 aPAP patients, reported a 78% objective response at M12 [29]. In the current series, the absence of a significant improvement after rituximab in the whole population and the 30% objective response were disappointing. Numerous hypotheses may explain this discrepancy between both series. Firstly, the patients included in the current series were less hypoxemic (mean initial PaO_2_: 68 mmHg) than those studied by Kavuru et al. (PaO_2_: 54 mmHg) [29]. Interestingly, we observed that patients who improved had lower initial PaO_2_ than patients who did not improve (*p* = 0.07). Secondly, in the current series, patients who already received a specific therapy for aPAP were less prone to respond, although patients from the Kavuru et al. trial had received a mean of 5 whole lung lavage before inclusion [29]. Some of the patients from the present series may present irreversible interstitial lung disease or pulmonary fibrosis, although we were not able to identify them by CT scan [34].

Our study showed that the improvement of the D(A-a)O_2_ was delayed after rituximab infusion, as it was only evidenced at M12 while it was absent at M6. Again, our results are discordant with Kavuru et al. who showed an improvement of D(A-a)O_2_, PaO_2_, pulmonary function tests and CT-scan from M6 [29]. Such a delayed efficacy of rituximab is not reported in other autoimmune diseases such as autoimmune hemolytic anemia or ANCA associated vasculitis. This delayed effect could be due to 1) the time needed to restore a normal alveolar macrophagic activity, that may be delayed from circulating monocyte activity and 2) to the time needed to evidence a functional effect of an improved alveolar surfactant clearance [35].

We cannot exclude that rituximab was not responsible for the improvement observed in 4 patients at M12, since spontaneous improvement has been reported in up to 30% of cases in the literature. Spontaneous improvement is mostly observed in patients with PaO_2_ > 70 mmHg, D(A-a)O_2_ < 40 mmHg and with higher DLCO [13, 17, 19, 36]. In the current series, mean initial PaO_2_ in the responder group was 64 mmHg, D(A-a)O_2_ was 42 mmHg, and DLCO was 49%.

This study has obvious limits such as its uncontrolled and retrospective nature and the relatively small number of patients studied. However, all French patients that received rituximab for aPAP were included in this study and therefore the 30% in intention to treat rate of response is a real life evaluation of rituximab efficacy in this very rare disease.

## Conclusions

This retrospective study does not support rituximab as a second line therapy for patients with refractory aPAP. Since whole lung lavage and inhaled GM-CSF are already very effective for this rare disease, the evaluation of rituximab as a first line therapy is unlikely due to its unconfirmed slowly developing effect.
